# Causation of Oxidative Stress and Defense Response of a Yeast Cell Model after Treatment with Orthodontic Alloys Consisting of Metal Ions

**DOI:** 10.3390/antiox11010063

**Published:** 2021-12-28

**Authors:** Vito Kovač, Matic Bergant, Janez Ščančar, Jasmina Primožič, Polona Jamnik, Borut Poljšak

**Affiliations:** 1 Faculty of Health Sciences, University of Ljubljana, Zdravstvena pot 5, 1000 Ljubljana, Slovenia; kovacv@zf.uni.lj.si; 2 Department of Environmental Sciences, Jozef Stefan Institute, Jamova 39, 1000 Ljubljana, Slovenia; matic.bergant@ijs.si (M.B.); janez.scancar@ijs.si (J.Š.); 3 Department of Dental and Jaw Orthopedics, Medical Faculty, University of Ljubljana, Hrvatski trg 6, 1000 Ljubljana, Slovenia; jasminaprimozic@gmail.com; 4 Biotechnical Faculty, University of Ljubljana, Jamnikarjeva ulica 101, 1000 Ljubljana, Slovenia; polona.jamnik@bf.uni-lj.si

**Keywords:** orthodontic appliance, metal ion release, oxidative stress, ROS, antioxidative defense

## Abstract

Misaligned teeth have a tremendous impact on oral and dental health, and the most efficient method of correcting the problem is orthodontic treatment with orthodontic appliances. The study was conducted to investigate the metal composition of selected orthodontic alloys, the release of metal ions, and the oxidative consequences that the metal ions may cause in the cell. Different sets of archwires, stainless steel brackets, and molar bands were incubated in artificial saliva for 90 days. The composition of each orthodontic material and quantification of the concentration of metal ions released were evaluated. Metal ion mixtures were prepared to determine the occurrence of oxidative stress, antioxidant enzyme defense system, and oxidative damage to proteins. The beta titanium alloy released the fewest metal ions and did not cause oxidative stress or protein damage. The metal ions from stainless steel and the cobalt-chromium alloy can cause oxidative stress and protein damage only at high concentrations. All metal ions from orthodontic alloys alter the activity of antioxidant enzymes in some way. The determined amounts of metal ions released from orthodontic appliances in a simulated oral environment are still below the maximum tolerated dose, and the concentrations of released metal ions are not capable of inducing oxidative stress, although some changes in antioxidant enzyme activity were observed at these concentrations.

## 1. Introduction

The number of patients seeking orthodontic treatment for malocclusions is constantly increasing. Depending on the type and severity of malocclusion, various treatment options are available, including fixed orthodontic appliances. The parts of fixed orthodontic appliances, including brackets, archwires, and bands, are made of various alloys that are meant to be durable, strong, resistant, and most importantly, biocompatible. However, the oral environment, with its constantly changing pH, temperatures, and biological and enzymatic compositions, is a stressful environment for any orthodontic appliance [1]. According to the American Board of Orthodontics, treatment with fixed appliances lasts approximately 24 months [2]; this raises the question of the safe use of such appliances. It is reported that glossitis, gingivitis, contact stomatitis, multiform erythema, and gingival hypertrophy may occur during orthodontic treatment, which could be related to the toxic effects of metal ions released by fixed orthodontic appliances [3].

Stainless steel, nickel-titanium (Ni-Ti), cobalt-chromium-nickel (Co-Cr-Ni), and β-titanium (β-Ti) are the most commonly used alloys for fixed orthodontic appliances. Nickel (Ni) has been described as the most allergic compound among orthodontic alloys [4], closely followed by Cr [5]. Titanium (Ti) is also widely used, not only in dentistry but also in other medical fields and also cosmetics. In addition, the most common metals in orthodontic alloys (Ni, Cr, Co, Fe, Ti) have been shown to cause toxic or biological side effects [6,7]. Although biocompatibility concerns arose over the years due to intraoral corrosion and tribocorrosion of various alloys, such as stainless steel, cobalt-chromium, and nickel-titanium, resulting in the increased release of ions with potentially toxic consequences, no consistent results were obtained to date. When evaluating scientific data, the oral environment, especially saliva, also has an impact on metallic materials as it is itself an electrolyte [8]. In vitro evaluation of the release of metal ions from orthodontic alloys is usually performed in artificial saliva, with an emphasis on mimicking in vivo conditions in terms of pH, temperature, and saliva composition [9].

Electrochemical corrosion reactions at the surface of biomaterials are redox reactions in which surface metals are oxidized to metal cations and metal oxides, which in turn can be released into the biological system [10]. Overproduction of reactive oxygen species (ROS), either endogenous or from exogenous sources, alters the redox balance between ROS and the cellular antioxidant system. This results in oxidative stress, which damages DNA, proteins, and lipids and has serious consequences. The best-known mechanism for the generation of ROS by metal ions is the Fenton reaction, in which transition metal ions react with hydrogen peroxide (H_2_O_2_) to produce a very reactive hydroxyl radical (^•^OH). Another metal ion ROS generating reaction is the Haber–Weiss reaction, in which a reduction of a metal ion occurs, and molecules of superoxide radical (O_2_^•−^) and H_2_O_2_ form ^•^OH [11]. Some metal ions have the redox capacity to generate oxidative stress via the Fenton or Haber–Weiss reaction, but they indirectly enhance the production of radicals by oxidizing glutathione, inducing NADPH oxidases, and affecting enzyme binding sites. Fe, Cr, Cu, Co, Ni belong to the transition metals [12] and are considered redox-active metals, i.e., they can transfer electrons between substrates, generating free radicals from the Fenton and Haber–Weiss reactions [13]. Iron can exist in ferrous (+III) and ferrous (+II) forms, where ferrous ions react with oxygen to form superoxide radicals [14] or generate hydroxyl radicals via the Fenton reaction [15]. Nevertheless, iron is also an essential cofactor for many enzymes of the electron transport chain as well as a cofactor for many catalases and peroxidases of the antioxidant defense [16]. Cr, Co, Ni, and certain metal ions can also participate in the Fenton-like reaction instead of iron [17]. Chromium occurs biologically in trivalent (Cr[III]) and hexavalent (Cr[VI]) forms, with the hexavalent form considered toxic but efficiently reduced to lower oxidation states and detoxified by chelators in vivo [18]. The reduction of hexavalent Cr produces reactive oxygen species such as superoxide and hydroxyl radicals, further unbalancing the redox scale [19]. Cobalt is another metal that uses the Fenton reaction to form reactive oxygen species [20]. Unlike other transition metals, nickel produces little ROS, but the mechanism of the Fenton reaction is the same [21]. The oxidation of Ni(II) to Ni(III) could be the cause of ROS formation. Molybdenum is an essential element in many functional enzymes of respiratory pathways and oxidative stress defense [22,23].

Under oxidative stress conditions, DNA, protein, and lipid damage occurs [24]. Xia et al. [25] suggest that increased oxidant levels in the cells initially trigger an antioxidant defense response, followed by inflammation and consequently cytotoxicity if the stress signal is strong enough. Thus, the first cellular response to increased oxidant concentrations is the induction of the cellular antioxidant defense system. To maintain redox balance, a complex endogenous antioxidant defense system is established in cells consisting of enzymatic antioxidants such as superoxide dismutase (SOD), catalase (CAT), peroxiredoxin (PRDX), glutathione peroxidase (GPx), glutathione reductase (GR), and thioredoxin reductase (TrxR) and endogenous non-enzymatic antioxidants such as glutathione (GSH) as well as exogenous vitamin C, carotenoids, and flavonoids [26]. The progression of oral diseases such as periodontitis, an inflammation of the gingival tissues, is closely associated with oxidative stress [27]. Markers of oxidative stress are also demonstrated in other periodontal diseases [28].

In this study, two topics were investigated: first, the release of metal ions from orthodontic appliances and the metal composition of orthodontic alloys, and second, the activation of the enzymatic antioxidant defense system and oxidative damage to proteins as a result of cell treatment with metal ions. Parts of orthodontic appliances (wires, brackets, and molar bands) made of different alloys (stainless steel, Ni-Ti, β-Ti, and Co-Cr) were incubated in a simulated oral environment for 90 days, and the amount of metal ions released was recorded over the period. The metal composition of each orthodontic alloy was also determined, and the data obtained were used to prepare metal ion mixtures further. In our previous study [7], we investigated the possible adverse effects that metal ion mixtures of different orthodontic alloys may have on a yeast cell model, highlighting the aspect of cytotoxicity and the formation of ROS. It was found that certain metal ion mixtures can cause cytotoxic effects and oxidative stress at certain concentrations. Since oxidative stress defense mechanisms are activated before the stress occurs, the focus here was on observing the activity of cellular enzymatic antioxidants deployed at metal ion concentrations ranging from 1 µM to 1000 µM. To assess oxidative damage to proteins, the carbonyl content of proteins was used as a marker.

## 2. Materials and Methods

### 2.1. Orthodontic Material Preparation

In the study, eight different types of samples were analyzed. Among them, six samples were different types of wires: stainless steel (Damon 0.016 × 0.25, Ormco, Brea, CA, USA), nickel-titanium (BioStarter 0.016”, Forestadent, Pforzheim, Germany and rematitan super elastic 0.016” Dentaurum, Ispringen, Germany), titanium-molybdenum (rematitan SPECIAL, Dentaurum, Ispringen, Germany), chromium-cobalt (Elgiloy 0.036” Rocky Mountain Orthodontics, Denver, CO, USA and remaloy 0.036” Dentaurum, Germany), and the remaining two were stainless steel brackets (Discovery Dentaurum, Ispringen, Germany) and stainless steel bands (W-Fit Form Forestadent, Pforzheim, Germany). A detailed description of all the samples is provided in Table 1. Both the upper and lower sections of each type of archwire were analyzed. In addition, a set of 24 brackets and 4 molar bands were tested. Each part of an apparatus (wire, bracket, and band) had the surface measured using a digital caliper. All materials were sterile before the beginning of the study.

To simulate oral conditions, artificial saliva was prepared using an aqueous solution of NaCl, KCl, CaCl_2_·2H_2_O, NaH_2_PO_4_·2H_2_O, Na_2_S·9H_2_O, and urea in concentrations of 400 mg/mL, 400 mg/mL, 960 mg/L, 690 mg/L, 5 mg/L, and 1000 mg/L, respectively. All reagents were of high purity and purchased from Merck (Darmstadt, Germany). Ultrapure water (Milli-Q, 18.2 MΩ cm) obtained from a Direct-Q 5 Ultrapure water system (Millipore, Watertown, MA, USA) was used for the solution preparation. The medium was then adjusted to a pH of 6.7–6.8, resembling neutral pH in the oral cavity. A WTW 330 pH (Weilheim, Germany) meter was employed to determine the pH.

Teflon beakers were used to minimize blanks and to prevent the absorption of metals on the walls of the containers during the experiment. To each beaker, 250 mL of artificial saliva were added along with the sample of parts of fixed orthodontic appliances (either two sets of wires, 24 brackets, or 4 molar bands). To control the blanks arising from the chemicals and the potential leaching of metal ions from the beakers used, 250 mL of artificial saliva were added into the Teflon beaker and analyzed along with the samples throughout the experiment. Blank values were subtracted from the determined concentrations of elements released from orthodontic appliances into the artificial saliva. All the experiments were prepared in duplicate. Samples were incubated in a dust-free incubator at 37 °C for 90 days. They were gently shaken to distribute the released metal ions into the leaching solution evenly. Sampling was carried out at seven different time points (t_1_ = 12 h, t_2_ = 24 h, t_3_ = 48 h, t_4_ = 7 days, t_5_ = 30 days, t_6_ = 60 days, and t_7_ = 90 days). Before the sampling procedure, each Teflon beaker was gently turned upside down two times to ensure the even distribution of elements in the sample volume. From each beaker, 3 mL of sample were taken, acidified with 6 µL of supra pure nitric acid, and stored at −20 °C until the analysis.

#### Metal Release and Alloy Constitution Analysis

To determine the metal composition of samples, approximately 10 mg of each sample were completely digested in 5 mL of appropriate acid by heating the solution at 90 °C. Stainless steel samples were digested in aqua regia, all titanium-based alloys in a mixture of HNO_3_, HF, and HCl (4:2:1 volume ratio), and Elgiloy and remaloy alloys in pure hydrochloric acid. Total metal ion concentrations in the digested samples and concentrations of metal ions released in the artificial saliva were determined by inductively coupled plasma mass spectrometry (ICP-MS) on an Agilent 7700x ICP-MS instrument (Tokyo, Japan), using matrix-matched standards for calibration. ICP-MS measurement parameters are presented in Table S1 of the supplementary material. Measurement uncertainty for all elements analyzed was better than ±3%.

Since there are no certified reference materials available for the determination of trace elements in orthodontic appliances, the accuracy of the determination of metal ions in the analyzed samples was assessed by the spike recovery test. For this purpose, bracket samples were spiked with known amounts of the elements analyzed before the digestion process, and the analytical procedure was applied. For checking the accuracy of the determination of the concentrations of metal ions released into artificial saliva, the samples collected 90 days after incubation were spiked with known amounts of metals and concentrations determined by ICP-MS. Good agreement between the theoretically calculated and measured concentrations (differences did not exceed ±5%) was obtained, confirming the accurate determination of total metal concentrations and the concentrations released into artificial saliva by ICP-MS. Blank samples of artificial saliva were analyzed along with the samples investigated throughout the experiment.

### 2.2. Orthodontic Metal Ion Solutions and Yeast Metal Treatment

To simulate orthodontic alloys, high-purity salts of FeCl_3_·6H_2_O, CrCl_3_·6H_2_O, NiCl_2_·6H_2_O, and CoCl_2_·6H_2_O, as well as titanium and molybdenum standards for AAS (TraceCERT, Merck, Darmstadt, Germany), were used. Final concentrations of 0.2 M stock solutions of metal mixtures were prepared with sterile ddH_2_O and had their pH adjusted to 7. Mixtures were also sonicated in an ultrasound bath to ensure sterile conditions. A detailed description of each metal ion concentration is represented in Table 1.

### 2.3. Preparation of Yeast Culture and Metal Treatment

*S. cerevisiae* wild type yeast strain BY4742 (EUROSCARF, Oberursel, Germany) was grown until the early stationary phase in a liquid yeast extract-peptone-glucose medium ((2% (*w*/*v*) glucose, 2% (*w*/*v*) peptone, and 1% (*w*/*v*) yeast extract (Merck, Darmstadt, Germany)) at 28 °C with 220 RPM constant shaking. After reaching the desired density, the yeast culture was transferred into PBS buffer (Merck, Darmstadt, Germany).

Yeast cultures were exposed to metal ion mixtures in concentrations of 1 μM, 10 μM, 100 μM, and 1000 μM. The untreated yeast culture served as a control sample. Treatment lasted 24 h in an incubator shaker at 28 °C and 220 RPM.

### 2.4. Reactive Oxygen Species (ROS) Level Determination

Detection of intracellular ROS level was performed using the fluorescent dye 2′,7′-dichlorofluorescein diacetate (H_2_DCF-DA). After the metal treatment, cells were washed twice with 50 mM potassium phosphate buffer, and the dye was added in a final concentration of 10 µM. An incubation time of 30 min in the dark and another washing step were employed before the dye fluorescence (ex/em = 488/520 nm) was measured with a Varioskan LUX microplate reader (Thermofisher, Waltham, MA, USA). To determine cell viability, cell suspensions were properly diluted with PBS to a dilution of 10^−6^ and plated on solid YPD plates. After 48 h incubation at 28 °C, yeast colonies formed on the solid YPD plates and were counted. The number of formed yeast colonies was expressed as colony forming units (CFU). The results were expressed as the fluorescence divided with values for the cell viability to obtain more accurate results.

### 2.5. Enzymatic Antioxidants Activity Determination

#### 2.5.1. Cell Lysate Preparation

Yeast cell samples were collected after 24 h metal treatment, centrifuged for 5 min at 4000 RPM and 4 °C, and washed twice with PBS. The cell pellet was resuspended in lysis buffer (0.05 M Tris–HCl pH 8 with protease inhibitor (Roche, Basel, Switzerland)) followed by glass bead homogenization. Cell lysates were centrifuged at 20,000× *g* and 4 °C for 20 min. The protein concentration of the obtained supernatant was determined using the Bradford assay [29].

#### 2.5.2. Superoxide Dismutase (Sod) Activity

Determination of SOD activity is based on the autoxidative ability of pyrogallol at alkaline pH [30]. When pyrogallol oxidates, a yellow product (purpurogallin) is formed, and the color change can be spectrophotometrically monitored at 420 nm absorbance, but in the presence of SOD, the rate of color change is depleted. The assay measures activities of all SOD types (Cu-Zn-SOD, Mn-SOD, and EC-SOD). When the final concentration of 0.3 mM pyrogallol is added to the wells with 50 mM cacodylic acid, 1 mM DTPA, and the enzyme sample, the color change rate at 420 nm is observed every 15 s for 5 min. More SOD is present in the sample, when the lesser the color change was observed. Results are expressed as the percentage activity of the SOD enzyme after treatment, compared to the enzyme activity of the untreated control sample.

#### 2.5.3. Catalase (CAT) Activity

CAT activity was determined by the rate of hydrogen peroxide removal, which can be observed as a decrease in absorbance at 240 nm. Lysate samples were diluted at 1:50 with 50 mM phosphate buffer. In a well, phosphate buffer, the diluted sample and 10 mM H_2_O_2_ were combined. The decrease in absorbance at 240 was monitored every 20 s for 5 min. Results are expressed as the percentage activity of the CAT enzyme after treatment, compared to the enzyme activity of the untreated control sample.

#### 2.5.4. Glutathione Peroxidase (GPx) Activity

GPx activity was determined with an adapted method from Smith and Levander [31]. Samples were mixed with a stock solution of 6.5 mM EDTA-Na_2_, 1.3 mM NaN_3_, 0.5 mM NADPH, 2.5 mM GSH, and 1.7 U/mL GR in 65 mM PBS. For the reaction to begin, 250 μM H_2_O_2_ were added. Absorbance change was measured every 15 s at 340 nm. Results are expressed as the percentage activity of the GPx enzyme after treatment, compared to the enzyme activity of the untreated control sample.

#### 2.5.5. Glutathione Reductase (GR) Activity

Measurement of GR activity was adapted from Glippa et al. [32] with small adaptations. The assay was based on the GR’s ability to convert oxidized glutathione (GSSG) to reduced glutathione (GSH) by using NADPH as a substrate. Formed GSH reacts with the 5,5′-dithiobis (2-nitrobenzoic acid) (DTNB) to generate a yellow-colored product. The change in the absorbance was measured after 3–6 min at 412 nm. Results are expressed as the percentage activity of the GR enzyme after treatment, compared to the enzyme activity of the untreated control sample.

#### 2.5.6. Thioredoxin Reductase (TrxR) Activity

The method from Smith and Levander [31] was slightly modified for TrxR activity evaluation. First, the reagent composing of 10 mM EDTA-Na_2_, 5 mM DTBN, 240 μM NADPH, and 0.2 mg/mL bovine serum albumin in 100 mM PBS was prepared. Then, lysate samples were separated into two groups: the first group was treated with 5% ethanol; the second group was treated with 5% ethanol containing 1.47 mM inhibitor auranofin. By adding the mixed reagent to samples, changes in absorbance at 412 nm were observed every 15 s for 5 min. Results are expressed as the percentage activity of the TrxR enzyme after treatment, compared to the enzyme activity of the untreated control sample.

#### 2.5.7. Peroxiredoxin (PRDX) Activity

PRDX activity was determined according to the method proposed by Ali and Hadwan [33]. Lysate samples were mixed with 2.1 mM 1,4-dithio-DL-threitol, 10 mM NaN_3_, 50 mM phosphate buffer, and 2,1 mM H_2_O_2_. After 3 min, the reaction was stopped by adding the titanium reagent (0.1% TiCl_4_ in 20% H_2_SO_4_). Due to residual H_2_O_2_, the concentration of newly formed peritanic acid could be assessed spectrometrically at 405 nm. Results are expressed as the percentage activity of the PRDX enzyme after treatment, compared to the enzyme activity of the untreated control sample.

#### 2.5.8. In-Gel Enzyme Activity

Zymograms of SOD and CAT were performed according to Weydert and Cullen [34]. The SOD gel assay was conducted on 12% non-denaturing polyacrylamide gel with a 5% stacking gel. At least 150 μg of the protein sample were used for each sample and loaded onto the gel. Samples were subjected to electrophoresis conditions for 3 h at 40 mA and 4 °C. Gels were stained for 20 min with SOD stain (2.43 mM NBT, 28 mM TEMED, and 0.14 M riboflavin 5′-phosphate in 50 mM PBS) and then washed twice with dH_2_O. Washed gels were placed in fresh dH_2_O and left under fluorescent light for at least 30 min or until distinctive bands appeared. Gels were washed three more times and were imaged. The same electrophoresis procedure was applied for CAT, but CAT and GPx gel assays were performed on 7.5% gels. Before staining, CAT gels were incubated in 0.003% H_2_O_2_ for 10 min, then washed twice with dH_2_O. The 2% ferric chloride and 2% potassium ferricyanide were prepared separately and used to stain the CAT gels. As soon as the bands appeared, the gel was washed extensively, and an image of the gel was taken. The band intensity of each gel image was determined by ImageJ, image-analysis software (Java-based image processing software, National Institutes of Health, Bethesda, MD, USA), and the activity of the treated samples was compared to the untreated control.

### 2.6. Oxidative Protein Damages

The reaction between 2,4-dinitrophenylhydrazine (DNPH) and protein carbonyl groups was employed to assess oxidative damage on proteins. Following the proposed method of Mesquita et al. [35], we combined our lysate sample with 10 mM DNPH and after a 10 min incubation, 6 M NaOH were added. Absorbance at 450 nm was obtained after 10 min and was compared between the sample containing protein and the sample where the lysate was substituted with buffer solution. Results are expressed as a percentage of protein oxidative damage after certain metal treatment, compared to the untreated control sample.

### 2.7. Statistical Analysis

A graphical presentation and statistical analysis were performed with GraphPad Prism (version 8.02 for Windows, GraphPad Software, La Jolla, CA, USA). A single-factor analysis of variance (One-way ANOVA) and Dunnett’s post hoc test were used to determine the differences between results. Results are expressed as means with 95% confidential intervals. Statistical significance was set at *p* < 0.05.

## 3. Results

### 3.1. Metal Ion Release

The metal compositions (Fe, Ni, Cr, Co, Ti, and Mo) of each part of the orthodontic appliances are presented in Table 1. Damon stainless steel archwires (Ormco, Brea, CA, USA), Discovery stainless steel brackets (Dentaurum, Ispringen, Germany), and W-Fit Form stainless steel bands (Forestadent, Pforzheim, Germany) had roughly the same composition of Fe, Ni, and Cr. Among them, only Discovery stainless steel brackets contained Mo (Mo = 1.95%). Both Ni-Ti archwires, rematitan super elastic (Dentaurum, Ispringen, Germany), and Biostarter (Forestadent, Pforzheim, Germany), included in the present study, had a very similar metal composition, Ni ≈ 73%, and Ti ≈ 26%. The two Co-Cr-Ni archwires, Elgiloy (Rocky Mountain Orthodontics, Denver, CO, USA), and remaloy (Dentaurum, Ispringen, Germany) differ from each other in every measured metal ion content, and only Elgiloy contained Mo (Mo = 2.65%). The rematitan SPECIAL Ti-Mo archwire (Dentaurum, Ispringen, Germany) was composed of Ti = 91.81% and Mo = 8.18%.

**Figure 1:**
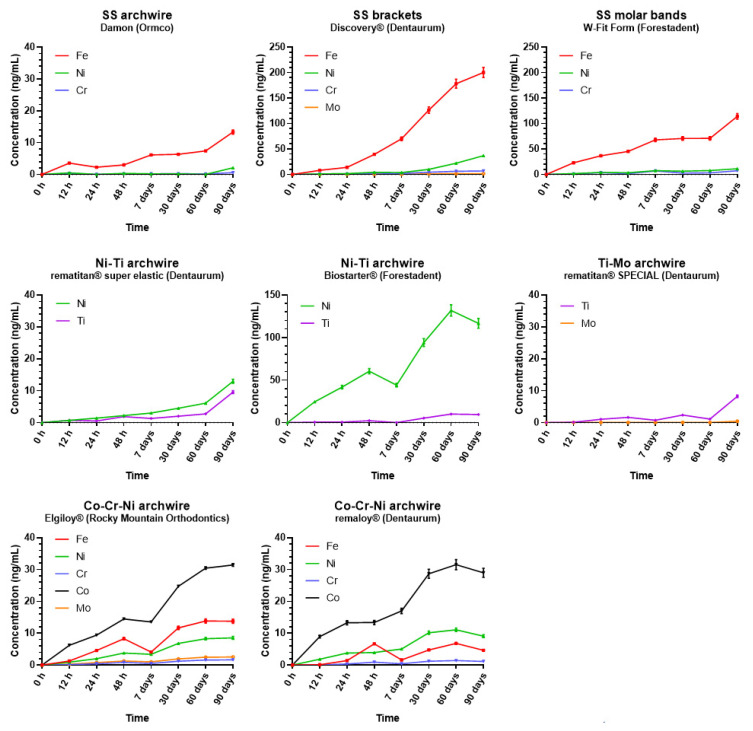
The release of metal ions from different parts of fixed orthodontic appliances into artificial saliva at different time points during the 90-day observational period. The results represent the average concentration of two parallel samples with standard deviations.

The release of metal ions from orthodontic appliances into artificial saliva (expressed in ng/mL) during the 90-day monitoring period is shown in Figure 1, while the corresponding metal ion concentrations are given in the supplementary material (Table S2).

As evident from Figure 1, in both stainless steel brackets and molar bands, Fe release was dominant over the other metals with a concentration of ≈200 ng/mL in the case of brackets and ≈114 ng/mL for molar bands at t_7_. Similarly, the amount of Fe released from stainless steel archwire steadily increased until the end of the study (Fe ≈ 13.3 ng/mL), while Ni and Cr concentrations released from them were near zero at all time points except at t_7_, when an increase in their concentrations was observed (≈2.1 ng/mL and ≈0.6 ng/mL, for Ni and Cr, respectively).

Regarding the Ni-Ti archwires, the same amount of Ti was released from both Ni-Ti archwires examined (Ti ≈ 9.5 ng/mL), while higher amounts of Ni were released (≈13 ng/mL and ≈116 ng/mL for Ni Dentaurum and Ni Forestadent, respectively). In the case of Ti-Mo archwires, Mo was below the limit of detection, except at t_7_, when its concentration was 0.45 ng/mL. Ti released from Ti-Mo archwires remained constant, approximately 1.5 ng/mL, during the entire observational period with a small peak, detected at the last time point (Ti ≈ 8.3 ng/mL). Metal release from both Cr-Co-Ni archwires increased gradually during the observational period. The concentrations of Cr, Co, and Ni detected were similar for both Elgiloy and remaloy archwires. Of note, a different concentration of Fe release (FeRemaloy ≈ 4.6 ng/mL, FeElgiloy ≈ 13.7 ng/mL) and the absence of Mo in the Remaloy archwire were detected. At t_7_, both Cr-Co-Ni archwires experienced stagnation in metal ions’ release with concentrations of ≈1.5 ng/mL, ≈30 ng/mL, and ≈9 ng/mL for Cr, Co, and Ni, respectively.

### 3.2. Intracellular Oxidation Level

**Figure 2:**
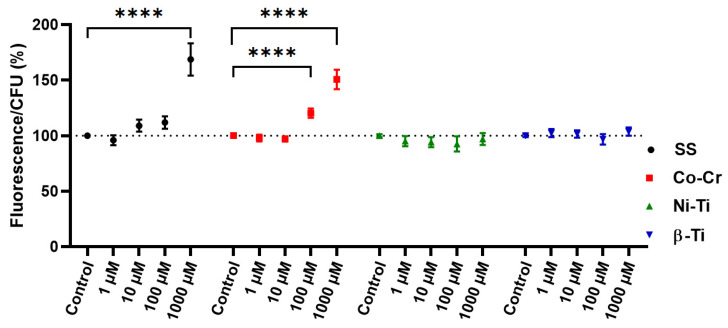
Ratio between the intensity of the fluorescence signal and CFU after treatment of yeast cells with different metal ion mixtures and concentrations. The percentage ratio of fluorescent dye intensity and the yeast CFU value were used to compare the intracellular oxidation of the treated viable cells to the untreated control sample, which was set at 100%. Means with the 95% confidence interval are shown, and the statistically different measurements are labeled with * (* *p* < 0.05, ** *p* < 0.01, *** *p* < 0.001, and **** *p* < 0.0001).

The ROS marker used to assess the intracellular oxidation level, H_2_DCFDA, is hydrolyzed intracellularly and converted to fluorescent DFC in the presence of ROS [36]. There is a correlation between the CFU count and the fluorescence intensity of the dye, since only metabolically active cells can hydrolyze the dye into a fluorescent product [37,38]. Valiakhmetov et al. [39] showed that under stress conditions, the population of viable ROS generating cells responds proportionally with the death of the cell population, implying that fluorescence intensity alone is not sufficient as an indicator of ROS generation and must be coupled with an assay for cell viability [40]. Figure 2 shows the relationship between the intensity of the fluorescent signal and the yeast cell CFU count. A significant increase in the ROS generation, as well as the cytotoxicity, was observed when treating yeast cells with a 1000 µM concentration of SS and 100 µM and 1000 µM Co-Cr concentration. When treating yeast cells with any other metal mixture or concentration, there was no observed difference between the treated and the untreated control cells.

### 3.3. Enzymatic Antioxidative Defense

The ability of enzymatic antioxidant defenses to remove excess oxygen radicals and their products prevents the development of oxidative damage of molecules. For this reason, the enzymatic antioxidative defense system was assessed by measuring the activity of superoxide dismutase, catalase, glutathione peroxidase, glutathione reductase, peroxiredoxin, thioredoxin reductase. As an increase in protein or mRNA expression of antioxidant enzymes does not necessarily mean an increase in activity, both spectrophotometric enzymatic assays and native gel electrophoresis methods were used to assess enzymatic antioxidative defenses.

**Figure 3:**
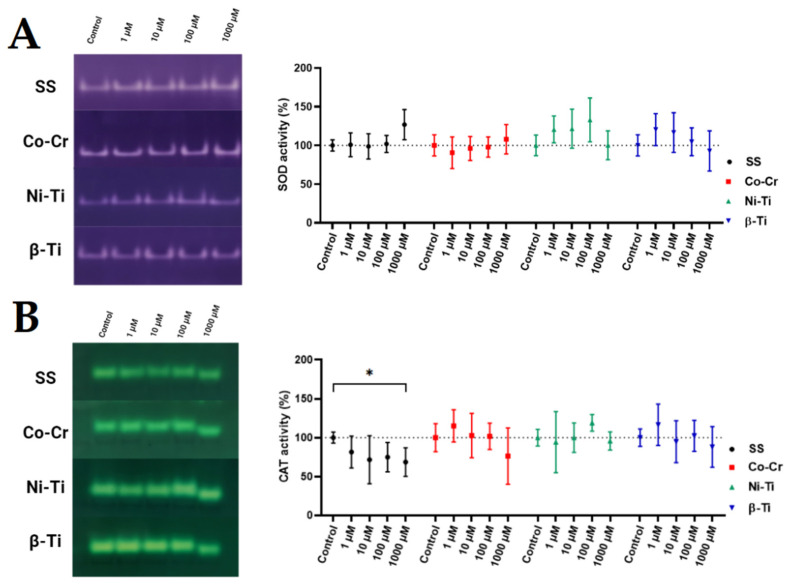
In-gel antioxidative activity of SOD (**A**) and CAT (**B**) enzyme after metal ion mixture treatment. The most representative images are shown (*n* = 5). Statistically different results in the graph are labeled with * (* *p* < 0.05).

In-gel antioxidant activity of SOD enzyme (Figure 3) did not show any statistically significant differences between metal treated and untreated control samples, even though some differences in the mean values could be observed. When treating yeast cells with any 1000 µM metal ion concentrations, a shift in the CAT band position was observed, while a statistically significant decrease in CAT activity was observed only in 1000 µM SS treated cells.

**Figure 4:**
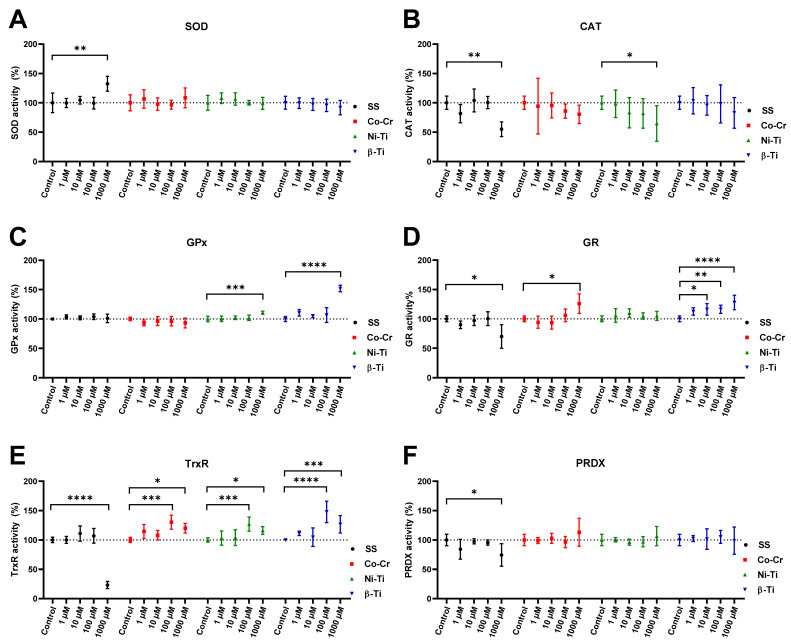
Change in antioxidant enzyme activities when yeast cells are treated with increasing concentrations of different metal ion mixtures, compared to the untreated control. Graph (**A**) shows SOD, (**B**) shows CAT, (**C**) shows GPx, (**D**) shows GR, (**E**) shows TrxR, and (**F**) shows PRDX activity. The untreated sample was set at 100%, and the means with 95% confident interval are shown. Statistically different results are labeled with * (* *p* < 0.05, ** *p* < 0.01, *** *p* < 0.001, and **** *p* < 0.0001).

Figure 4 shows the activity of the following enzymes: SOD, CAT, GPx, GR, PRDX, and TrxR, measured spectrophotometrically. The activity of the enzyme SOD increased only when the cells were treated with a concentration of 1000 µM SS. On the other hand, treatment of the cells with different concentrations and metal mixtures did not result in any changes in SOD activity. The CAT activity assay gave more variable results, as the CAT activity decreased noticeably with an increasing metal concentration, with the activity decreasing significantly only when treated with a 1000 µM SS and 1000 µM Ni-Ti concentration. GPx activity was not affected by metal treatment, except that a significant increase in GPx activity was detected when cells were treated with 1000 µM Ni-Ti and 1000 µM β-Ti concentrations. Cell lysates from 1000 µM SS treatment had a decreasing effect on the GR activity, whereas 1000 µM Co-Cr and 10, 100, and 1000 µM β-Ti treatment significantly increased the GR activity. Again, there was a decrease in the activity of 1000 µM SS cell lysate in the case of TrxR activity, but in all other lysates of cells treated with 100 µM and 1000 µM concentrations, TrxR activity was statistically increased. In the case of PRDX activity, only a decrease in activity was observed in the cell lysate from treatment with 1000 µM SS.

### 3.4. Protein Oxidative Damage

**Figure 5:**
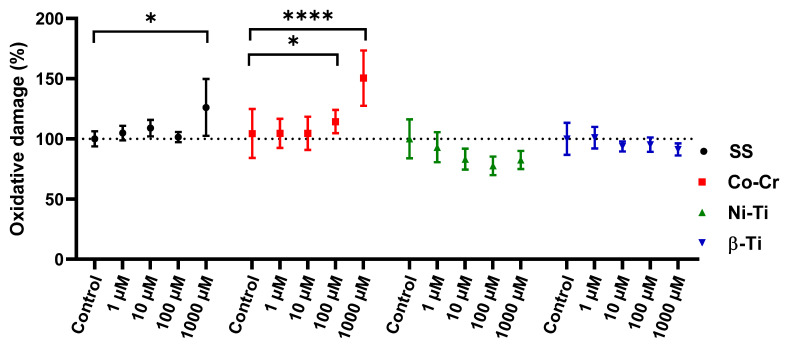
Protein carbonyl content as a result of oxidative protein damage as a result of cell treatment with various metal ions. The control sample was set at 100%, and the means with 95% confident interval are shown. Statistically different results are labeled with * (* *p* < 0.05, ** *p* < 0.01, *** *p* < 0.001, and **** *p* < 0.0001).

When proteins are oxidized, stable carbonyl groups are produced on the aminoacid side chains, thus making them an ideal marker for determining the oxidative damage to proteins (Figure 5). A statistical difference in protein oxidative damage between the untreated control and the one treated with metal mixtures was observed in the case of 1000 µM SS, 100 µM, and 1000 µM Co-Cr concentrations. Cell lysates from NiTi and β-Ti treatment did not show any changes in protein carbonyl content, compared to the untreated control sample.

## 4. Discussion

### 4.1. Topic: Metal Ion Release in a Simulated Environment

The present study evidenced an increase in metal ions’ concentrations released from different parts of fixed orthodontic appliances over 90 days of exposure to artificial saliva. Although an increase in the measured cumulative concentrations was detected over the observational period, all the measured ions’ concentrations were still far below the recommended dietary intake levels. This sense of safety may be underestimated due to non-toxic concentrations of metals inducing biological effects in oral cells [41]. Hypersensitivity to metals, such as nickel, must also be a prime concern when treating orthodontic patients [42]. Overall, the quantity of leached ions increased during the study with minor fluctuations, probably due to minor sampling or measuring errors. Of note, we aimed to assess the concentrations of the five most common metal ions present in alloys of orthodontic appliances. Therefore, it cannot be excluded that other metal ions were also released from the samples. By using pH-neutral artificial saliva, we evaded the possible pH fluctuation that occurs in the oral cavity due to food and biofilm decomposition [43]. This indicates that higher ion release values could be obtained in vivo. Metallic alloys are in use in many medicinal applications, ranging from cardiovascular orthopedic, craniofacial, otorhinology, and dentistry fields [44]. Their biocompatibility makes them inert and allows them to perform the intended requirements while not causing any adverse effects to the host [45]. These ideal properties are endangered by the occurrence of tibocorrosion [46] by which released particles and metal cations from alloys could provoke adverse local tissue reactions [47,48]. According to Brantley and Eliades [49], there are mainly four types of orthodontic alloys in common orthodontic use with the following compositions: stainless steel (Cr = 17–20%, Ni = 8–12%, Fe to balance), cobalt-chromium (Co = 40%, Cr = 20%, Ni = 15%, Mo = 7%, Fe = 16%, other metals = 2%), nickel-titanium (Ni = 55%, Ti = 45%), and β-titanium (Ti = 77.8%, Mo = 11.3%, other metals = 10%) [50,51]. The composition of our orthodontic alloys is slightly different from the presented ones, but there are a lot of different alloy types currently on the market, each having changes in its composition [52].

Ni-Ti archwires were obtained from two manufacturers, and different metal ions releases were detected, despite the same metal composition. Ti concentrations in both archwires were nearly equal after 90 days of exposure but increased steadily throughout the study. While titanium should form an oxide layer to protect against corrosion [53], its increase in the medium is a sign of the deterioration of this protective surface. When observing rematitan super elastic Ni-Ti archwire (Dentaurum) ion release patterns, the concentration of Ni and Ti ions increased in the same manner; the Biostarter Ni-Ti archwire (Forestadent) had ten times more metal ions released than the other Ni-Ti wire. Even though the composition of alloys is the same among different manufacturers, they are not necessarily of the same quality. In addition, surface topography, processing, and finishing techniques in archwire production play a major part in corrosion resistance since surface roughness and surface defects are the major corrosion sites [54]. According to The European Committee for Standardization devised via EN 1811:2011, nickel released from products must not exceed 0.5 µg/cm^2^/week [55]. After just 7 days, Ni-Ti_Biostarter_ released 2568 µg/cm^2^/week nickel, and after 13 weeks (90 days), we obtained 0.501 µg/cm^2^/week ion release which is the same as the prescribed nickel limit. Because metal release rates were nonlinear for 90 days, a much higher release was obtained in the first week of exposure compared to the last week of exposure, emphasizing the meaning of the performed longitudinal study. Of all orthodontic alloys used in the study, only Co-Cr-Ni alloy metal release seemed to plateau, but since it happened at the end of the study, we cannot conclude that it happened because of metal saturation.

Ni was released in six times higher concentrations from Ni-Ti archwires than the stainless steel archwire, an effect also observed by Charles et al. [56] and Hussain et al. [57], where the nickel release of Ni-Ti-archwire was 4.85 ng/mL compared to the stainless steel archwire 0.41 ng/mL ion release. This was expected, as the nickel amounts to more than 55% of the Ni-Ti orthodontic alloy, compared to 15% in the SS alloy. Contrary to our findings, Suárez et al. [58] compared corrosion resistance of stainless steel, Ni-Ti, and titanium archwire. They found out that the stainless steel alloy was the least corrosive resistant, meaning that the protective role of Cr_2_O_3_ oxide film in stainless steel is lesser than TiO_2_ from the titanium-containing alloy.

The smallest cumulative amounts of metal ions released were from β-Ti archwires, since this type of archwire has more titanium content to form the protective oxide layer and also a fair share of Mo to form the MoO_3_ oxide layer [59]. The Ti-Mo archwire released almost two times fewer Ti metal ions compared to both Ni-Ti archwires, whereas the Mo ion release was negligible until the last sampling time point.

According to the reported upper intake limits (UL), the highest levels of dietary intake that impose no threat to the general population for the examined metal ions are UL_Ni_ =1 mg/d, UL_Fe_ = 45 mg/d, UL_Mo_ = 2 mg/d, and UL_Ti_ = 1.1 mg/d [60]. For Cr, an upper intake limit has not yet been established, but its recommended daily intake is 200 µg/d [60]. Comparing our findings with the UL, even when considering the cumulative concentrations, none of the examined released metal ions exceeded the prescribed daily intake concentrations. Interestingly, after comparing released ion concentrations for each orthodontic material with the alloys’ metal composition, it appears that there is no correlation. Stainless steel brackets, for example, contain 56% of Fe, but the relative percentage of the released Fe metal ions was much higher than that at 80%. A similarity to our findings was previously reported by Hwang et al. [61]. In their study, Hussain et al. [62] concluded that the ion release is not dependent on the alloy ion amount but rather on the nature of the alloy and its manufacturing process.

Several previous in vitro studies aimed to assess the release of metal ions from orthodontic appliances leading to contradicting results. Straightforward comparisons are not possible due to the different study designs, in terms of the material analyzed, immersion media, and analytical equipment. Mikulewicz et al. [63] reported that an orthodontic appliance made of stainless steel after a period of 30 days of immersion in artificial saliva leached 2382 ng/mL Fe, 573 ng/mL Ni, and 101 ng/mL Cr. Even after adding up the concentrations of metal ions released from stainless steel brackets, molar bands, and archwires after 90 days of exposure, the results, although similar in the ratios between specific metal ions concentrations, were significantly smaller (304.01 ng/mL Fe, 46.83 ng/mL, Ni and 13.87 ng/mL Cr) in comparison with that reported previously by Mikulewicz et al. [63]. The higher values evidenced by Mikulewicz et al. could be due to their specific study design that included constant shaking of the samples at 120 rpm over a period of 30 days whereas in our study samples were slightly shaken only before sampling. By this, we were able to measure the concentrations of leached metal ions only due to the pure diffusion of ions with no additional mechanical forces to enhance the ion release. Even higher values of Fe and Cr ions released from stainless steel orthodontic appliances were shown by Hwang et al. [61], while similar results were reported regarding the concentrations of Ni ions released from Ni-Ti archwires. Kuhta et al. [64] compared the Ni-Ti wire with stainless steel wire and gained similar results to this study for Ti. They also showed how acidic pH values impacted more ions being released. Ortiz et al. [3] also compared the metal release of titanium and stainless steel, but in the combination of brackets and bands, and also found that the titanium-containing alloy is more biocompatible than others.

The above-mentioned studies all used some sort of a combination of wire, brackets, and bands to determine metal ion release. In the present study, metal ion release from brackets, molar bands, and archwires were measured separately and in the case of archwires, also according to their material alloys composition. This could be regarded as a limitation of the study since it excluded the possible corrosive effect and related ions released due to different materials’ interactions. The additional mechanical aspect of bracket, band, and archwire friction should not be neglected because they can additionally contribute to the metal ions’ release [65]. Performing multi-element analysis with the ICP-MS, we were able to determine exactly which part of the orthodontic appliance accounted for the greatest ions release compared to the others and its release profile characteristic during the 90-day study. The number of separate parts tested would appropriately simulate the usual amount of which orthodontic appliances are composed in everyday clinical practice. When submerging orthodontic materials, previously mentioned studies did not account for the adsorption of positively charged ions to the negatively charged surface. To prevent this occurrence, the submersion medium should be acidified to provide an excess of H^+^ ions. In our study, to obtain a steady pH level of seven for 90 days, acidifying was inadequate. To our knowledge, this was the first study to use Teflon beakers as vessels for orthodontic material submersion, thus preventing any possible metal ion adsorption to the beaker surface and maintaining a constant pH level throughout the study.

Recently, Wendl et al. [66] tested ion release from several types of brackets, bands, and archwires, but only for Ni, Cr, Mn, and Co. This study does not directly apply to in vivo conditions but provides an important insight into how each orthodontic alloy reacts differently in a simulated environment. Although a 90-day study is quite representative of the use of archwires, it does not represent the simulation for the use of brackets and molar bands, which are subjected to the oral cavity for approximately two to three years.

Moreover, in vitro experiments are not able to simulate an actual in vivo environment fully. For example, corrosion is the main reason for ion release, and the scale of it depends on the formation of passive oxide layers on the surfaces of orthodontic materials. These oxide layers, Cr oxide or Ti oxide, effectively prevent corrosion [67] but are highly influenced by the changes in the oral environment, such as pH and temperature levels. In our study, a constant pH and temperature were retained to avoid a potential increase in ion release [64].

Obtained results of metal ion release enabled us to estimate the metal ion combinations and concentrations for further studies of oxidative stress causation and consequently involvement of the enzymatic antioxidative stress defense. Namely, such defense represents the first cellular response to increased oxidant levels [25].

### 4.2. Topic: Metal Ion Exposure, Oxidative Stress, and the Enzymatic Antioxidant Defense System Induction

There are not many studies dealing with ROS caused by dental or orthodontic alloys. In our study, we found an increased formation of ROS in Figure 2 only in two types of metal ion mixtures: stainless steel and cobalt-chromium. The simulation of nickel-titanium and β-titanium alloys, in the form of metal cations, did not lead to the formation of ROS. Similar results were obtained in a previous study [7]. Interestingly, the study by Spalj et al. [68] showed that Ni-Ti wire produced the highest amount of ROS while SS and β-titanium wire did not. In their further study, they showed that only high concentrations of Ni and TiO_2_ salt-induced the formation of ROS in gastrointestinal tract cell lines [69]. As in our study, Pallero et al. [70] also used a mixture of ions to simulate the alloy SS and evaluate the formation of ROS. They also confirmed that the metal mixture SS increases the levels of ROS in vascular smooth muscle cells, for which Fe, Cr, Ni, and Mo ions seem to be responsible. As for Co-Cr alloy, Salloum et al. [71] used Co and Cr ions separately to observe ROS production in macrophages. Only Co(II) ions induced oxidative stress in macrophages, while Cr(III) ions did not. On the other hand, previous studies show that Cr(II) also induces oxidative stress, but to a lesser extent than Co(II) [72,73]. The combined effect of using a Co-Cr metal ion mixture resulted in an increase in ROS in yeast cells at 100 µM and 1000 µM concentrations [72]. The addition of Cr(III), Fe(III), Mo(III), or Ni(II) ions to mouse embryo fibroblasts and liver HepG2 cells increased the formation of ROS in a dose-dependent manner, which was first observed at concentrations as low as 100 µM [74].

**Figure 6:**
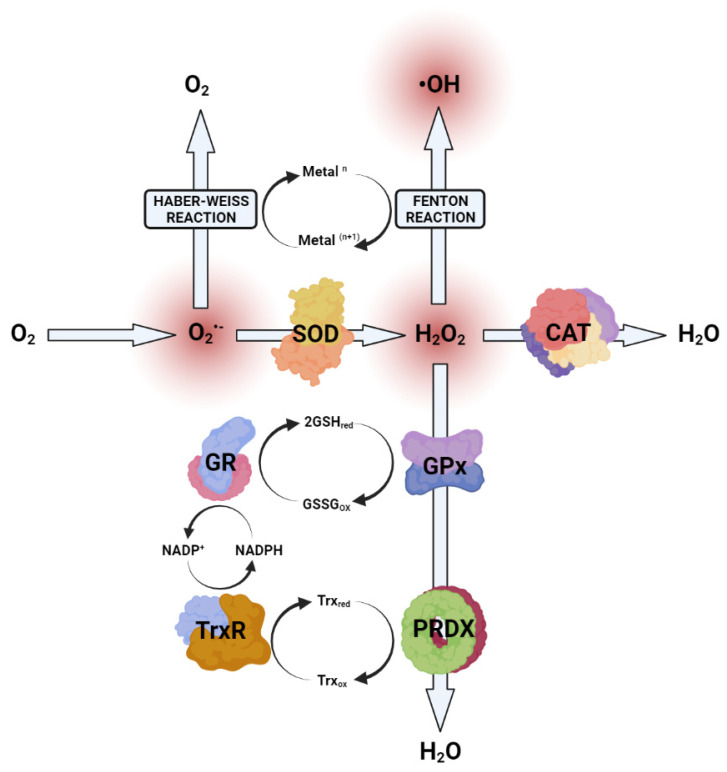
Generation of ROS and the involvement of the enzymatic antioxidative defense system. O_2_^•−^ superoxide anion; ^•^OH hydroxyl radical; SOD superoxide dismutase; CAT catalase; GPx glutathione peroxidase; GR glutathione reductase; PRDX peroxiredoxin; TrxR thioredoxin reductase.

Each organism has an appropriate antioxidant defense system to cope with the overproduction of ROS and maintain redox homeostasis. Figure 4 shows the cell enzymatic antioxidants such as superoxide dismutase (SOD), catalase (CAT), glutathione peroxidase (GPx), glutathione reductase (GR), peroxiredoxinreductase (PRDX), and thioredoxins (Trx) used to suppress ROS overproduction. Non-enzymatic molecules such as glutathione also play a key role in antioxidant defense [75] but were not the subject of our study. The activity of the above enzymes is used to assess the occurrence of increased ROS levels because the cell’s enzymatic antioxidant defense system responds earlier to an elevated level of ROS as it can be detected as a result of oxidative damage. The most important in vivo defense enzyme is considered to be SOD, which converts O_2_^•−^ to H_2_O_2_, which is then further converted to H_2_O by CAT. The reduction in H_2_O_2_ is also mediated by the glutathione peroxidase (GPx) protein family, which uses low molecular weight thiols such as glutathione (GHS) as a catalyst for the conversion of H_2_O_2_ to H_2_O [76]. Because SOD, CAT, and GPx are metalloenzymes, metal ions are implemented as cofactors and as such are a necessity for the enzyme activity. Copper, zinc, and manganese are essential for SOD, iron for CAT, and selenium for GPx activity [77]. Several thiol-containing enzymes, thioredoxin reductase (TrxR) and peroxiredoxin (PRDX), are also involved in the removal of H_2_O_2_ [78], but require a reducing agent in the form of NADPH. Thus, NADPH and its oxidized form NADP^+^ are essential cofactors of the enzymes TrxR and GR (Figure 6).

Because of its easy accessibility, rapid growth, and genetic relevance to human disease, *Saccharomyces cerevisiae* is a suitable model organism for oxidative stress studies [79]. Through evolution, all eukaryotic organisms, from yeast to vertebrates, possess the same antioxidant enzymes [80], making the yeast *S. cerevisiae* particularly important for simulating the eukaryotic cellular processing of ROS. *S. cerevisiae* in the stationary phase strongly resemble multicellular organisms because the yeast cells in the stationary phase transit from the fermentative to mitochondrial respiratory metabolism for energy and growth. Second, the cells in the stationary phase are in a quiescent state in which they are neither dividing nor preparing to divide. The last reason is that any damage that has occurred is accumulated and must be prevented or repaired, as the potential masking or mitigation of damage by new divisions or synthesis is diminished [81]. The transition to respiration naturally increases the production of ROS and evokes modulation of the antioxidant system, making stationary cells more susceptible to oxidative stressors [82]. Stationary yeast cells are permanently exposed to endogenously produced ROS, which attacks the same cell all the time, and oxidative damages are accumulated. If we were to use yeast cells in the exponential phase, the main source of energy would come from glycolysis, and the cells would be exposed to the stressor only for a short time before starting to divide again, leading to potentially false results [83]. Furthermore, the budding of cells forming new daughter cells also prevents the possible accumulation of damaged molecules in the mother cells [84]. Such exponentially growing cells rarely occur in the natural environment or human body; thus, extrapolation of obtained results from exponential phase cells to multicellular organisms would be misleading. When oxidative stress occurs, antioxidant defense mechanisms are theoretically activated to prevent potential oxidative damage. Since an increase in protein or mRNA expression of antioxidant enzymes does not necessarily mean an increase in activity, we used both spectrophotometric enzymatic assays and native gel electrophoresis to assess enzymatic defense. Treatment of yeast cells with Fe, Cr, and Ni ions, in the case of 1000 µM SS, increased the activity of SOD. This could be due to either excessive leakage of the SOD substrate, superoxide, from the electron transport chain due to mitochondrial damage or increased function of NADPH oxidase (NOX). The product of superoxide reduction by SOD should be the generation of H_2_O_2_, which should also trigger the increase of CAT, GPx, or PRDX, which was not confirmed in our study. Indeed, a decrease in the activity of CAT and PRDX was observed, whereas the activity of GPx did not change. One possible explanation is that H_2_O_2_, when generated, immediately enters the Fenton reaction with metal cations, especially Fe cations, forming a hydroxyl radical. This is evident to some degree when cells are treated with 1000 µM Fe, Ni, and Cr combinations, with the increase in ROS level, decreased cell viability, and increased protein carbonyl content. Further evidence is the absolute annihilation of TrxR activity [85], which converts NADP^+^ to NADPH, a general mediator of the detoxifying and antioxidant defense system [86]. When cells were treated with Co-Cr metal ion mixtures, no statistically significant difference in enzyme activity was observed, although a slight increase in SOD and PRDX was noticed. Again, CAT activity appears to decrease in a dose-dependent manner when yeast cells are treated with all-metal ion mixtures, even statistically significant in the case of SS and Ni-Ti. Atli et al. [87] observed that the inhibition of CAT is due to the binding of the metal ion to the enzyme—SH groups. Lower CAT activity in yeast cells was also observed by Bayliak et al. [88] and was due to inhibition by high substrate concentrations, as CAT activity is not linear with H_2_O_2_ concentration. The authors suggested that a critical oxidant concentration in the cells might be the cause for the observed results. When evaluating the CAT in-gel activity, conclusions could be drawn about the CAT inhibition at high concentrations of metal treatments. The higher electrophoretic mobility of the CAT enzyme (band) when exposed to 1000 µM metal concentrations could be a consequence of the potential oxidation of the catalase. This argument could be supported by the study of Rodríguez-Ruiz et al. [89], in which they found the same increase in band mobility due to increasing H_2_O_2_ treatment and potential oxidative damage.

It is emphasized that the concentrations of reduced, oxidized, and total glutathione are markers of the oxidative stress response [90]. Since the GPx enzyme uses GSH as a substrate, its activity should be closely related to the GSH/GSSG ratio. Metal ion mixtures of SS and Co-Cr did not affect GPx activity, but the Ni-Ti and β-Ti mixture increased the activity at 1000 µM concentrations. As for the activity of GR, which regenerates GSSG to GSH used for GPx activity, no link between these two enzymes could be established. According to Tandoğan and Ulusu [91], Ni^2+^ ions coexist with GSSG for binding the active site of the GR enzyme, inhibiting its activity. SS, Co-Cr, and Ni-Ti metal alloy mixtures contain different amounts of Ni^2+^ ions, but the Ni-Ti mixture did not change the activity of GR, while the Co-Cr mixture increased the activity, and the SS mixture decreased it. Compared to GPx, PRDX enzymes can reduce organic and inorganic H_2_O_2_ without using metal ions as cofactors [92] and often compete with GPx for H_2_O_2_ dissociation [93]. This was not observed in the presented study, as the activity of PRDX enzyme did not change, except when yeast cells were treated with a concentration of 1000 µM, where the activity was significantly inhibited. The inhibition of PRDX is supported by the fact that the TrxR enzyme is also inhibited at this specific concentration of the SS ion mixture, which means that the electron donor, thioredoxin (Trx), is absent for the reaction to occur.

Lazarova et al. [94] found an increase in SOD and CAT activity after 6 h of treatment with Cr ions. Feng et al. [95] also observed an increase in SOD and CAT activity, but only when cells were treated with low Cr concentrations and for a short time. Longer exposures and higher chromium concentrations inhibited enzyme activities, implying that antioxidant enzymes can only protect cells to a certain extent before the organism succumbs to the fate of oxidative stress. When macrophage cells are treated with Cr(III) ions for 12 h, there is a dose-dependent inhibition of CAT activity, apparently due to Cr ions replacing Fe in the active sites of the CAT enzyme [72].

A similar study was performed by Terpilowska et al. [74] by treating BAKB/3T3 and HepG2 cells with chromium, iron, nickel, and molybdenum. An increase in the intracellular ROS level and MDA concentrations were observed. The authors observed how each individual metal affected oxidative stress markers, but concluded that a mixture of two metals (e.g., chromium and iron) had an additive effect on oxidative stress generation. When the SOD, CAT, and GPx activity were evaluated, an increase in activity was observed at 100 µM and 200 µM ion concentrations, but a statistically significant decrease was observed only with increasing metal concentrations above 400 µM. The same effect was observed by Kalaivani et al. [96] for nickel and by Siddiqui et al. [97] for molybdenum. In a cell-free study, treatment of the CAT enzyme with concentrations of 0–50 µM Cr(III) ions increased the activity of the CAT enzyme, but when the 50 µM concentration is exceeded, the enzyme activity decreases, probably due to interactions between the active site of CAT and the excess of chromium ions [98].

The release of Co and Cr ions from alloys is already documented in many studies on hip replacement implants [99,100,101], but also to a lesser extent on orthodontic alloys. When macrophages were treated with Co(II), Cr(VI), and Cr(III) ions for 24, 48, and 72 h, Cr(VI) ions induced the expression of SOD, CAT, and GPx proteins, whereas Cr(III) and Co(II) had no effect. A negligible effect of Cr(III) and Co(II) on the enzyme CAT was also found by Fleury et al. [102]. Both Co(II) and Cr(III) appeared to be cytotoxic to macrophages at concentrations of 24 ppm and 250 ppm, but only Co(II) was able to induce the production of ROS and protein carbonylation [71]. The difference between the toxicity of the two Cr ions might lie in the accessibility of the ions to enter the cell. While Cr(VI) can easily pass through the anion channels, Cr(III) can only be taken up into the cell via phagocytosis [103]. On the other hand, Magone et al. [104] suggested the possibility of Cr(III) oxidation to Cr(IV) to ensure cell membrane passage. In the present study, Co(II) chlorides and Cr(III) chlorides were used to simulate Co-Cr alloys because Co(III) and Cr(VI) are rapidly reduced to lower oxidation states under psychological conditions [105].

Studies on the effects of orthodontic alloys on antioxidant enzyme activity are perplexing. After 1 and 24 weeks of orthodontic treatment, the activity of SOD, CAT, and PRDX were not observed in unstimulated total saliva, but a decrease in the activity of SOD and CAT was observed in stimulated total saliva [106]. A research study by Bandeira et al. [107] showed that stainless steel bands induced the expression of *SOD1* and *PRDX1* genes but not the *GPX1* gene.

The determination of carbonylated proteins can be used as a biomarker for metal-induced oxidative stress since the reaction is irreversible [94]. As expected, oxidative damage to proteins occurs only when excessive amounts of ROS are produced, so the graphs from Figure 2 and Figure 5 look similar. The same effect was observed in other studies [71,72]. The protein oxidation of 100 µM and 1000 µM concentrations for Co(II) and Cr(III) in the study [72] agreed with our result when the Co-Cr mixture was treated with the same concentrations. Lazarova et al. [94] treated yeast cells with 1000 µM concentrations of Cr ions for 6 h and observed an increase in ROS production and consequently an increase in protein carbonyl content. When yeast cells were treated with NiTi and β-Ti in the presented study, no oxidative stress was observed.

An interesting observation was made by Scarcello et al. [108] when they used Fe particles instead of solid materials. Immediately after Fe particles were synthesized, they formed a protective oxide layer on their surface [109]. The generation of ROS occurred even in the presence of the oxide layer for Fe particles, but SS powder did not generate ROS. This study is also in agreement with Fagali et al. [110]. They both emphasize that direct contact with the material (surface chemistry) and not indirect contact (degradation products—Fe^3+^ ions) causes ROS generation. In contrast to Fe-containing alloys, Co-Cr alloys have a direct and indirect effect on ROS values [111], mainly due to the immediate Co dissolution in body fluids, thus changing the protective oxide layer to chromium and molybdenum oxide [9].

In general, Co-Cr alloys are the toughest, stiffest, and more wear-resistant compared to Fe-based (stainless steel) and Ti-based alloys [112], but the alloy SS offers the best price-performance ratio, and the Ti-based alloys have their specific strength and are also considered to be the best biocompatible as well as the most corrosion-resistant [113,114]. As evidenced in this study, the β-Ti alloy wire arch released the least metal ions during the 90-day incubation, followed by the SS, Ni-Ti, and Co-Cr alloy. As expected, the SS brackets and molar bands used in this study released more metal ions due to their larger combined surface area. To hypothesize the amount of metal ions that could be released from an entire orthodontic appliance consisting of 2 archwires, 24 brackets, and 4 bands, the concentration after 90 days gives a total of 400 ng/mL when only the SS alloy is used. These results reflect only passive ion diffusion from orthodontic alloys and do not take into account possible physiological or biological changes that could increase the release of metal ions. Regarding the composition of the orthodontic alloy, the combined release of metal ions was comparable to the 10 µM metal ion mixtures used to treat yeast cells. Concentrations of 100 µM and 1000 µM were also used to evaluate possible effects that a higher metal ion release could have. When yeast cells were treated with either 1000 µM SS or 100 µM and 1000 µM Co-Cr mixtures, we observed a positive correlation between the occurrence of oxidative stress and oxidative protein damage. No unilateral interpretation could be made when assessing the overall enzymatic antioxidant defense system.

When the increased activity of the SOD enzyme was observed, it was expected that the activity of the CAT or GPx enzyme would also increase due to H_2_O_2_ production in the dis-mutase reaction, but this increase was not observed. The mechanism of enzyme activation is very complex, and only the evaluation of multiple antioxidant enzyme activities can provide an estimate of the extent of oxidative stress occurrence in cells. Since we used metal ion mixtures in the present study, we were not able to identify a specific metal responsible for inducing oxidative stress or affecting the activity of antioxidant enzymes. It is worth noting that the changes in enzyme activity are transient and time-dependent, as the defense response is very dynamic, but the damages caused are the final products that accumulate in the cell.

## 5. Conclusions

Orthodontic appliances should be biocompatible for short- and long-term use to ensure safety and so as not to harm the patient during orthodontic treatment. The release of metal ions from different orthodontic alloys during a 90-day exposure to artificial saliva did not exceed the recommended upper limits for daily metal intake. The β-Ti alloy was considered the most biocompatible alloy, as its metal release was the lowest among the other orthodontic alloys tested. Depending on the composition of the metal alloy, metal ion mixtures were prepared for the treatment of yeast cells. The metal ion mixtures of stainless steel and cobalt-chromium induced oxidative stress at high (non-physiological) concentrations as well as the oxidation of proteins in the form of carbonylation. A diverse antioxidant enzyme defense system was also induced when cells were treated with metal ions at higher concentrations. Although metal ions can cause oxidative stress and oxidative damage, as well as induce enzymatic antioxidant defenses, the risk of adverse effects on patient health from treatment with fixed orthodontic appliances is considered low because we have shown that concentrations of released metal ions from selected orthodontic materials do not exceed the concentration required to induce oxidative stress and cause cellular protein damage. It is possible that locally high metal concentrations may be present due to corrosion or metal degradation. Therefore, it is important that the antioxidant defense system is activated and effectively protects against possible increased ROS and oxidative stress-induced damage.

## Figures and Tables

**Table 1:** Orthodontic materials used in the study with their corresponding metal composition in weight percentages (%).

Component	Type	Specification	Parts in the Sample	Combined Surface (cm^2^)	Fe (%)	Ni (%)	Cr (%)	Co (%)	Mo (%)	Ti (%)
Archwire	Stainless steel	Damon 0.016 × 0.25 Ormco, Brea, CA, USA	2	5.104	58.3	14.6	27.1	<0.1	<0.1	<0.1
	Ni-Ti	Biostarter 0.016” Forestadent, Pforzheim, Germany	2	4.172	<0.1	73.8	<0.1	<0.1	<0.1	26.2
	Ni-Ti	rematitan super elastic 0.016” Dentaurum, Ispringen, Germany	2	4.149	<0.1	73.2	<0.1	<0.1	<0.1	26.8
	Ti-Mo	rematitan SPECIAL 0.032” Dentaurum, Ispringen, Germany	2	6.846	<0.1	<0.1	<0.1	<0.1	8.18	91.8
	Co-Cr-Ni	Elgiloy 0.036” Rocky Mountain Orthodontics, Denver, CO, USA	2	9.476	6.12	19.9	21.6	49.7	2.65	<0.1
	Co-Cr-Ni	remaloy.036” Dentaurum, Ispringen, Germany	2	9.217	1.42	26.1	19.4	53.2	<0.1	<0.1
Brackets	Stainless steel	Discovery Dentaurum, Ispringen, Germany	24	14.478	55.4	17.8	24.9	<0.1	1.95	<0.1
Molar bands	Stainless steel	W-Fit Form Forestadent, Pforzheim, Germany	4	9.881	57	18.2	24.9	<0.1	<0.1	<0.1

## Data Availability

Data is contained within the article and supplementary materials.

## Supplementary Materials

The following are available online at https://www.mdpi.com/article/10.3390/antiox11010063/s1, Table S1: ICP-MS operating parameters for determination of element concentrations, Table S2: Release of metal ions from stainless steel, Co-Cr-Ni, Ni-Ti, β-Ti archwire, stainless steel brackets, and molar bands.
