# Supplementary material for: Causation of Oxidative Stress and Defense Response of a Yeast Cell Model after Treatment with Orthodontic Alloys Consisting of Metal Ions
Source: Antioxidants (Basel). 2021 Dec 28;11(1):63. doi: 10.3390/antiox11010063 (PMC8772795; doi:10.3390/antiox11010063)
Supplement: Supplementary file 1 [file antioxidants-11-00063-s001.zip › antioxidants-1498221-supplementary.pdf]

Supplementary material

Table S1: ICP-MS operating parameters for determination of element concentrations.

Parameter	Type/Value
Sample introduction	
Nebuliser	Miramist
Spray chamber	Scott
Skimmer and sampler	Ni
Plasma conditions	
Forward power	1550 W
Plasma gas flow (Ar)	15.0 L/min
Carrier gas flow (Ar)	1.05 L/min
Dilution gas flow (Ar)	0.10 L/min
Collision gas flow (He)	4.5 mL/min
Oct bias	-100 V
Cell entrance	-100 V
Cell exit	-150 V
Deflect	-75 V
Plate bias	-150 V
Sample uptake rate	0.3 mL/min
Data acquisition parameters	
Isotopes monitored	^{47}Ti, ^{52}Cr, ^{56}Fe, ^{59}Co, ^{60}Ni, ^{95}Mo
Isotopes of internal standards	^{72}Ge, ^{103}Rh

Table S2: Release of metal ions from stainless steel, Ni-Ti and Ti-Mo archwire

Stainless steel archwire (Damon - Ormco, USA)	Ti (ng/mL)	Cr (ng/mL)	Fe (ng/mL)	Co (ng/mL)	Ni (ng/mL)	Mo (ng/mL)
12 h	<0.1	0.36	3.59	<0.01	0.50	<0.05
24 h	<0.1	<0.05	2.28	<0.01	<0.05	<0.05
48 h	<0.1	0.23	3.00	<0.01	0.36	<0.05
7 days	<0.1	0.22	6.14	<0.01	0.21	<0.05
30 days	<0.1	0.25	6.36	<0.01	0.23	<0.05
60 days	<0.1	0.17	7.41	<0.01	<0.05	<0.05
90 days	<0.1	0.58	13.36	<0.01	2.11	<0.05
Ni-Ti archwire (rematitan® super elastic - Dentaurum, Germany)	Ti (ng/mL)	Cr (ng/mL)	Fe (ng/mL)	Co (ng/mL)	Ni (ng/mL)	Mo (ng/mL)
12 h	0.73	<0.05	<0.1	<0.01	0.73	<0.05
24 h	0.50	<0.05	<0.1	<0.01	1.43	<0.05
48 h	1.87	<0.05	<0.1	<0.01	2.22	<0.05
7 days	1.30	<0.05	<0.1	<0.01	3.01	<0.05
30 days	/	/	/	/	/	/
60 days	2.74	<0.05	<0.1	<0.01	6.10	<0.05
90 days	9.59	<0.05	<0.1	<0.01	12.97	<0.05
Ni-Ti archwire (Biostarter® - Forestadent, Germany)	Ti (ng/mL)	Cr (ng/mL)	Fe (ng/mL)	Co (ng/mL)	Ni (ng/mL)	Mo (ng/mL)
12 h	0.52	<0.05	<0.1	<0.01	24.59	<0.05
24 h	0.50	<0.05	<0.1	<0.01	41.81	<0.05
48 h	2.28	<0.05	<0.1	<0.01	60.62	<0.05
7 days	<0.1	<0.05	<0.1	<0.01	44.23	<0.05
30 days	5.27	<0.05	<0.1	<0.01	94.03	<0.05
60 days	10.07	<0.05	<0.1	<0.01	131.93	<0.05
90 days	9.53	<0.05	<0.1	<0.01	116.66	<0.05

Table S2 (continued): Release of metal ions from Co-Cr-Ni archwire, stainless steel brackets and molar bands

Ti-Mo archwire (rematitan® SPECIAL - Dentaurum, Germany)	Ti (ng/mL)	Cr (ng/mL)	Fe (ng/mL)	Co (ng/mL)	Ni (ng/mL)	Mo (ng/mL)
12 h	<0.1	<0.05	<0.1	<0.01	<0.05	<0.05
24 h	1.04	<0.05	<0.1	<0.01	<0.05	<0.05
48 h	1.64	<0.05	<0.1	<0.01	<0.05	<0.05
7 days	0.76	<0.05	<0.1	<0.01	<0.05	<0.05
30 days	2.39	<0.05	<0.1	<0.01	<0.05	<0.05
60 days	1.12	<0.05	<0.1	<0.01	<0.05	<0.05
90 days	8.26	<0.05	<0.1	<0.01	<0.05	0.45
**Co-Cr-Ni archwire (Elgiloy® - Rocky Mountain Orthodontics, USA)**	**Ti (ng/mL)**	**Cr (ng/mL)**	**Fe (ng/mL)**	**Co (ng/mL)**	**Ni (ng/mL)**	**Mo (ng/mL)**
12 h	<0.1	0.16	1.27	6.21	0.90	0.27
24 h	<0.1	0.42	4.59	9.43	2.02	0.78
48 h	<0.1	0.85	8.29	14.49	3.79	1.28
7 days	<0.1	0.52	4.08	13.54	3.37	1.02
30 days	<0.1	1.23	11.68	24.74	6.80	1.95
60 days	<0.1	1.58	13.84	30.46	8.29	2.47
90 days	<0.1	1.66	13.77	31.45	8.55	2.54
**Co-Cr-Ni archwire (Remaloy® - Dentaurum, Germany)**	**Ti (ng/mL)**	**Cr (ng/mL)**	**Fe (ng/mL)**	**Co (ng/mL)**	**Ni (ng/mL)**	**Mo (ng/mL)**
12 h	<0.1	<0.05	<0.1	8.96	1.86	<0.05
24 h	<0.1	0.34	1.42	13.27	3.77	<0.05
48 h	<0.1	0.92	6.67	13.38	3.94	<0.05
7 days	<0.1	0.42	1.62	17.02	5.06	<0.05
30 days	<0.1	1.22	4.74	28.68	10.14	<0.05
60 days	<0.1	1.40	6.80	31.56	11.07	<0.05
90 days	<0.1	1.15	4.66	28.98	9.09	<0.05

Table S2 (continued): Release of metal ions from Co-Cr-Ni archwire, stainless steel brackets and molar bands

Stainless steel brackets (Discovery® - Dentaurum, Germany)	Ti (ng/mL)	Cr (ng/mL)	Fe (ng/mL)	Co (ng/mL)	Ni (ng/mL)	Mo (ng/mL)
12 h	<0.1	0.82	8.27	<0.01	1.10	<0.05
24 h	<0.1	1.05	13.96	<0.01	1.72	0.15
48 h	<0.1	2.64	39.45	<0.01	4.19	0.70
7 days	<0.1	1.99	70.07	<0.01	3.89	0.47
30 days	<0.1	4.50	126.48	<0.01	10.07	1.25
60 days	<0.1	6.36	178.31	<0.01	22.29	1.66
90 days	<0.1	6.92	200.25	<0.01	37.03	1.83
Stainless steel molar bands (W-Fit Form Forestadent, Germany)	Ti (ng/mL)	Cr (ng/mL)	Fe (ng/mL)	Co (ng/mL)	Ni (ng/mL)	Mo (ng/mL)
12 h	<0.1	1.01	23.02	<0.01	1.85	<0.05
24 h	<0.1	3.73	36.85	<0.01	4.01	<0.05
48 h	<0.1	1.52	45.30	<0.01	3.45	<0.05
7 days	<0.1	7.01	67.99	<0.01	7.67	<0.05
30 days	<0.1	2.72	70.64	<0.01	6.59	<0.05
60 days	<0.1	3.13	70.84	<0.01	7.73	<0.05
90 days	<0.1	7.44	113.98	<0.01	11.31	<0.05
